# Bee Pollen as a Promising Agent in the Burn Wounds Treatment

**DOI:** 10.1155/2016/8473937

**Published:** 2016-05-18

**Authors:** Paweł Olczyk, Robert Koprowski, Justyna Kaźmierczak, Lukasz Mencner, Robert Wojtyczka, Jerzy Stojko, Krystyna Olczyk, Katarzyna Komosinska-Vassev

**Affiliations:** ^1^Department of Community Pharmacy, School of Pharmacy and Division of Laboratory Medicine in Sosnowiec, Medical University of Silesia in Katowice, Kasztanowa 3, 41-200 Sosnowiec, Poland; ^2^Department of Biomedical Computer Systems, Faculty of Computer Science and Materials Science, Institute of Computer Science, University of Silesia, Bedzinska 39, 41-200 Sosnowiec, Poland; ^3^Department of Clinical Chemistry and Laboratory Diagnostics, School of Pharmacy and Division of Laboratory Medicine in Sosnowiec, Medical University of Silesia in Katowice, Jednosci 8, 41-200 Sosnowiec, Poland; ^4^Department and Institute of Microbiology and Virology, School of Pharmacy and Division of Laboratory Medicine in Sosnowiec, Medical University of Silesia in Katowice, Jagiellonska 4, 41-200 Sosnowiec, Poland; ^5^Center of Experimental Medicine, Medics 4, Faculty of Medicine in Katowice, Medical University of Silesia in Katowice, 40-752 Katowice, Poland

## Abstract

The aim of the present study was to visualize the benefits and advantages derived from preparations based on extracts of bee pollen as compared to pharmaceuticals commonly used in the treatment of burns. The bee pollen ointment was applied for the first time in topical burn treatment. Experimental burn wounds were inflicted on two white, domestic pigs. Clinical, histopathological, and microbiological assessment of specimens from burn wounds, inflicted on polish domestic pigs, treated with silver sulfadiazine or bee pollen ointment, was done. The comparative material was constituted by either tissues obtained from wounds treated with physiological saline or tissues obtained from wounds which were untreated. Clinical and histopathological evaluation showed that applied apitherapeutic agent reduces the healing time of burn wounds and positively affects the general condition of the animals. Moreover the used natural preparation proved to be highly effective antimicrobial agent, which was reflected in a reduction of the number of microorganisms in quantitative research and bactericidal activity of isolated strains. On the basis of the obtained bacteriological analysis, it may be concluded that the applied bee pollen ointment may affect the wound healing process of burn wounds, preventing infection of the newly formed tissue.

## 1. Introduction

Wound healing, being the result of dynamic cooperation between many molecular factors, is a dynamic reaction whose undisturbed course enables restoring the continuity and functionality of damaged skin [1–3]. The process consists of 4 specific phases which smoothly proceed and change from one to the other even coexisting at times. The duration period of particular healing phases may vary depending on the type of the damage and possible coexistence of interfering additional factors, that is, the size and place of the damage, blood supply of the wound edges, cleanness of the wound, the degree of microbiological contamination, presence of necrotic tissue, and properly conducted healing management [1, 2, 4, 5].

The therapy of burn wounds may be properly conducted either by applying surgical methods or by topical application of therapeutic preparations. Besides contemporary, conventional treatment methods of thermal skin damage, apitherapy, which uses the therapeutic effect of standardized, pharmacologically active fractions obtained from bee products, is becoming more and more noticeable. Apitherapeutic agents have a beneficial effect on the skin condition, due to the reduction of water loss, and influence the reconstruction of the lipid barrier. One of the most frequently used apitherapeutic agents is bee pollen. This is a varied, natural product which is rich in such biologically active substances as amino acids, fatty acids, phytosterols, phospholipids, nucleic acids, carbohydrates, vitamins, mineral substances, enzymes, and coenzymes as well as phenolic compounds including phenolic acids and flavonoids [6–9]. The plethora of biologically active substances gives this natural raw material significant biotic properties such as antimicrobial, anti-inflammatory, immunomodulatory, or antioxidative activity [7, 10].

Such a high efficiency of this natural bee product with a significantly low risk of adverse reactions makes bee pollen a potentially optimal remedial factor in the therapy of local burn wounds [11, 12]. Therefore, the subject of this study was the assessment of efficiency and therapeutic usability of the bee pollen which has not been studied before.

Bee pollen, also flower pollen, is male reproductive organs produced by flowers of entomophilous plants. It is collected by worker bees, transported, and stored in beehives. It constitutes a basic ingredient in bee's nutrition used for current needs or stored for later period [13]. Bee pollen results from agglutination of flower pollen, nectar or honey, and bee's salivary substances [14].

Bee pollen treatment of topical, thermal damage of the skin was compared with the commonly applied pharmaceutical preparation such as silver sulfadiazine (SSD), which has many side effects.

AgSD not only may be responsible for the development of argyria and dysfunctions of liver, spleen, and kidney due to systemic accumulation of silver or determined by sulphadiazine presence, dermatitis, erythema multiforme rashes, and acute hemolytic anemia but also unfortunately could be responsible for cytotoxic effect on fibroblasts and keratinocytes [15, 16].

The clinical assessment of the treatment process of burn wounds was conducted. It concerned wound pathomorphology including the extent and depth of the burn, wound maceration, occurrence, and character of the exudate as well as the process of scar formation. The histopathological assessment of the burn wound epithelialization of the dynamics was done together with qualitative and quantitative assessment of particular microorganisms in tissue samples collected from beds of experimental burn wounds.

## 2. Material and Methods

### 2.1. Therapeutic Agents

The following therapeutic preparations were used: 1% silver sulfadiazine (SSD) (Lek, Poland), 0.9% NaCl (Polpharma), and bee pollen formulation. The analyzed bee pollen came from the apiary “Barć” in Kamienna. These are clean and ecological regions of Poland. In this apiary the European Dark Bee also known as Western Honey Bee is bred. The pollen was a composition of many pollens of various plants. Taking into account the location of the apiary, the dominating pollens came from such plants as oilseed rape (lemon-yellow color), shamrock (brown color), coltsfoot (bright yellow), common dandelion (bright orange), linden (bright green), or heather (red-yellow). Macroscopically, it was a multicolor blend of granules which were ground. 50 g of the ground pollen was added to 500 mL of 70% ethanol. The extraction of the solution was conducted for 4 weeks at room temperature. After that period, the solution underwent microfiltration. Next, the ethanol was distilled with vacuum evaporator. The result was dry matter which was used to prepare the bee pollen formulation (ointment containing 5% bee pollen ethanolic extract and 95% of petroleum jelly (according to Polish norm PN-R-78893)). The procedure was performed under general anesthesia according to the dosage regimen: atropine sulfate, 0.05 mg/kg body weight (Polfa Warszawa); ketamine hydrochloride, 3 mg/kg body weight (Biovet, Puławy); xylazine hydrochloride, 1 mg/kg body weight (Sandoz GMBH). Silver sulfadiazine was used in order to prolong the analgesic effect, 5 mg/kg body weight (Polpharma).

### 2.2. Tissue Material

The study protocol was approved by the Ethics Committee of the Medical University of Silesia. Two, 16-week-old, domestic pigs have been chosen as the useful experimental animals for the evaluation of wound repair because of many similarities of pig skin to human one. The usage of the limited number of experimental animals was consistent with validated animal model developed by Hoekstra et al. [17] in modification of Brans et al. [18]. The last mentioned pig model is based on the application of one experimental animal [18]. Moreover, in accordance with the guidelines of good laboratory practice for animal testing, the established principle is to use the minimum number of animals necessary to arrive at scientifically robust data and to ensure reliable data. Thus, the animals used in our study were bred and selected for the highest degree of genetic purity. This form of breeding purpose prevents genetic contamination and allows minimizing the number of animals necessary for the experiment, with very reliable results to be obtained.

Pigs were housed according to G.L.P. standards of Polish Veterinary Law. Each animal was inflicted with 18 skin burn wounds with equal gaps (9 wounds on each side along the line of the backbone). The size of each wound was identical, 1.5 cm × 3 cm. Totally, the wounds took about 2% of the surface of each animal's body subject to the experiment.

Burns were classified as 2nd-degree deep partial thickness burns. Animals were divided into two groups: control (C) and experimental ones (E). 36 dermal burns were inflicted. The wounds of animals in the control group were either untreated (subgroup C1) or treated with physiologic saline (subgroup C2).

The postburn wounds of the experimental group were also treated with SSD (subgroup E1) and with the bee pollen containing ointment (subgroup E2). The wounds in question were treated with mentioned substances twice a day, starting on the first day of the experiment. Three replications of biopsies were taken from the same wound of each animal, using surgical knife. Occlusive dressings were applied every 12 hours in all animals of all subgroups.

### 2.3. Clinical Study

Clinical observation was to assess the extent and depth of the burn, its maceration, and presence of necrotic tissue in it. Macroscopic reading of pathomorphological picture of the wound considered occurrence and intensification of typical symptoms of burn wounds: erythema, swelling, exudate, bleeding, and eschar. The process of granulation tissue formation together with the course of scar formation, ongoing on the burn wound surface, was also assessed.

### 2.4. Histopathological Study

The process of granulation, the type of the granulation tissue, intensification of swelling around the burn angiogenesis, and possible scarring of the wound were assessed. The microscopic picture of skin preparations included degree of the damage in the area and near the wound as well as the repair processes in next stages of the observation. Histopathological studies concerned the samples which were collected from burn wounds and from the adjacent, unchanged tissue in general anesthesia on 0, 3rd, 5th, 10th, 15th, and 21st day from the moment of inflicting the burn. After consolidation, tissues samples were collected form skin specimen in order to make histopathological preparations. The basic slides with samples were stained to achieve optical differentiation and verification of the elements of cell structure. Two different kinds of dyes were used: hematoxylin and eosin. Two histopathological preparations, which resulted from that process, underwent the microscopic assessment.

### 2.5. Microbiological Study

Microbiological study was performed from the material collected from the burn wounds on 0, 3rd, 5th, 10th, 15th, and 21st day of the experiment. In the case of quantitative study, the material was collected with a sterile swab stick from the burn wound surface of 1 cm^2^ and was subsequently put into the 10 cm^3^ of a sterile solution of 0.9% NaCl. This suspension of microorganisms served as the basis for a series of dilutions. Then, a 1 cm^3^ of the suspension was collected and spilt on the slide and dissolved in both the Mueller Hinton agar (MH), in order to assess the amount of bacteria, and Sabouraud agar, in order to assess the amount of fungi and mould. The material to microbiological purity test of the skin was simultaneously collected from the places where the burns were not inflicted. In the case of quantitative studies, the material was collected with AMIES transport medium with active carbon (HAGAMED, Poland), which was stored at 5°C up to the moment of performing microbiological tests (max. up to 2 hours). Simultaneously, the samples for microbiological purity test were collected from the skin of animals not taking part in the experiment. Microbiological diagnosis was conducted in accordance with the standards of National Committee for Clinical and Laboratory Standards [19]. The cultures were conducted on the following enrichment and differential media such as liquid media (Carbohydrate broth, an enrichment medium for aerobic bacteria) and solid media (blood agar, to enrich aerobic microorganisms and characterize the type of hemolysis; Mannitol Salt Agar (Chapman), to differentiate* Staphylococcus* spp.; MacConkey Agar, to differentiate* Enterobacteriaceae* species; Sabouraud Agar, to identify fungi; Agar D-Coccosel, to identify* Enterococcus faecalis*; Cetrimide Agar, to identify* Pseudomonas* spp.). The identification of isolated bacteria species was conducted by microscopic tests, culture tests, and commercial biochemical test API (bioMerieux, France). The growth promotion test was carried out with reference strains. The next stage of the test was to assess the amount of bacteria on 1 cm^2^ of the burn wound surface. Therefore, the material was collected from 1 cm^2^ of the wound which was then shaken in 10 cm^3^ of the sterile solution of physiological saline.

### 2.6. Data Analysis

In addition to the analytical methods mentioned above, the automatic measurement of the time constants was proposed. They concern the change rate analysis of the number of bacteria, fungi, or moulds in the wound. Therefore, the electrical-analog method, the inertial first-order object with delay, was suggested. For such a proposed model, the time constants for particular groups C1, C2, E1, and E2 were measured.

The microbiological data analysis was performed using Statistica 7.0 package (StatSoft, Cracov, Poland). The normality of distribution was verified with Kolmogorov-Smirnov test. Statistical differences between variables were verified by analysis of variance (ANOVA), followed by post hoc NIR test.

## 3. Results

### 3.1. Clinical Test Results

The clinical view of the wounds was compared on 3rd, 5th, 10th, 15th, and 21st day after burn infliction.

Differences in the clinical view of healing wounds were noticed on the 5th day of the observation. In the control subgroups, untreated wounds (C1), wounds treated with 0.9% NaCl solution (C2), and the study subgroup (E1) in which the wounds were treated with silver sulfadiazine, the erythema was observed to exceed the area of the burn wound. The skin surrounding the wound was very swollen with visible exudate. In the case of the wounds treated with the ointment with a 5% bee pollen, the subgroup (E2), the area of the wound was covered with a thin, flexible eschar accompanied by bleeding. On 10th and 15th day of the experiment, the untreated wound, in the control subgroup (C1), was covered with a hard, dry, and cracked eschar strongly adhering in the center. Under the eschar, there was a pink granulation tissue. During the same days, in the control subgroup, treated with 0.9% NaCl solution (C2), the burn wound was covered with a softened eschar with a small amount of serosanguineous exudate. In the experimental subgroup (E1) treated with silver sulfadiazine, the area of the wound was covered with a hard eschar and there was an erythema. The burn wounds of subgroup (E2), treated with the bee pollen ointment on the 10th day, were covered with a thin, flexible eschar with a visible granulation, while, on the 15th day, there was a clear epithelium being formed. The area of the wound decreased. The tissues surrounding the wound were characterized by a weak, atrophic inflammatory condition. On the 21st day of the observation, the clinical view was still significantly differentiated. In subgroup (C1) the untreated wounds were covered with a dry, cracked eschar. In subgroup (C2), the wounds, being constantly washed with 0.9% NaCl, were covered with an irregular eschar tightly adhering to the wound in its central part. After the eschar was removed mechanically, a pink granulation tissue without the features of epithelialization could be seen. The wound, treated with silver sulfadiazine (subgroup E1), was covered with a pink epithelium. The tissues surrounding the wound had no significant inflammatory features. The wound area did not decrease. The wounds, treated with the bee pollen ointment, in subgroup E2, were covered with a thick epithelium. The features of the healing process were strongly visible. Within the surrounding tissue there were not any signs of erythema or the ongoing inflammatory process.

### 3.2. Histopathological Test Results

**Figure 1:**
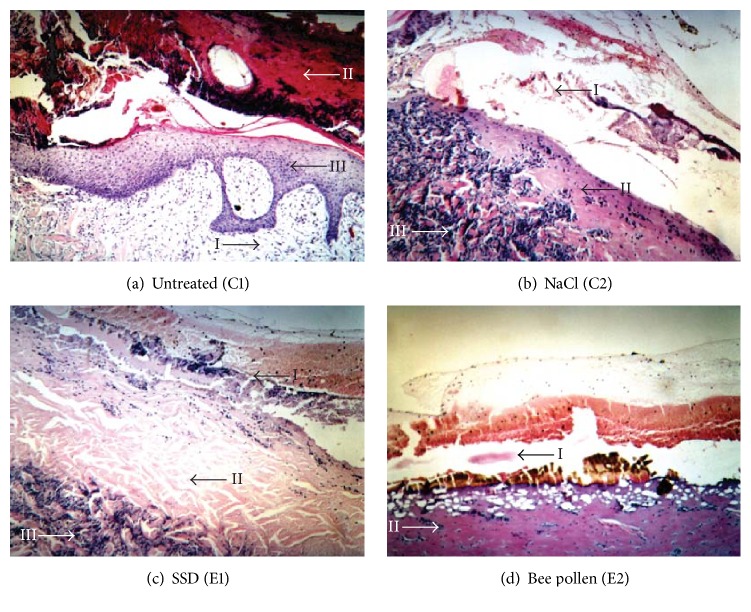
The picture of microscopic changes of skin samples collected from burn wounds on the 10th day of the experiment: (a) untreated (I: swollen inflammatory granulation tissue in the area of dermis, II: eschar with a slight bleeding, and III: visible, pink, and swollen granulation tissue); (b) washed with NaCl (I: petechial hemorrhages, loss of stratified squamous epithelium, II: coagulative necrosis, and III: massive lymphocytic infiltration); (c) treated with SSD (I: petechial hemorrhages, II: area of aseptic necrosis with many inflammatory infiltrations, and III: inflammatory infiltrations on the verge of necrosis); (d) treated with bee pollen (I: petechial hemorrhages, II: area of necrosis with many inflammatory infiltrations).

The histological view of wound healing of animals from all groups until the 5th day of the experiment were identical.  Figure 1 shows differentiated dynamics of repairing processes which occurred on the 10th day of the experimental healing process for all analyzed groups. Application of the bee pollen (E2) achieved its therapeutic effect on the 10th day of the experiment. The whole wound surface was filled with collagen fibers, which affected scar formation, and the stratified squamous epithelium was being created.

**Figure 2:**
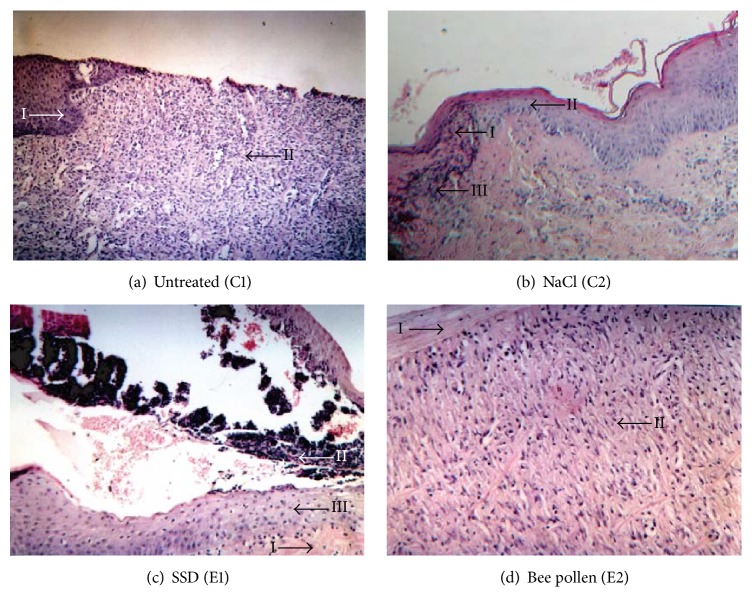
The microscopic changes of skin samples collected from burn wounds on the 21st day of the experiment: (a) untreated (I: regenerated stratified squamous epithelium on the sample edge, II: vessel-rich and cell-rich granulation tissue); (b) washed with NaCl (I: eschar, II: regenerated stratified squamous epithelium, and III: vessel-rich and cell-rich inflammatory granulation tissue); (c) treated with SSD (I: a slightly swollen dermis, II: eschar with petechial hemorrhages, and III: regenerated stratified squamous epithelium); (d) treated with bee pollen (I: connective tissue scar covered with epithelium and II: inflammatory granulation tissue with predominating collagen fibers).

On the 15th day of the observation, other changes in the histopathological view were observed. In the control subgroups C1 and C2 and in the E1 subgroup, a slow wound healing process in the phase of fibroplasia with the sustaining inflammation could be observed. In subgroup (E2), in which the wounds were treated with the ointment with 5% bee pollen extract, fibroplasia was significantly proceeding, while the present granulation tissue was covered with a regenerated epithelium. In the wound area there were no clear signs of inflammatory reaction. The regenerated stratified squamous epithelium was appearing on the wound edges together with existing inflammatory infiltrations in the histopathological view on the 21st day of the experiment in the case of the untreated wounds (subgroup C1). In case of wounds washed with 0.9% of NaCl (subgroup C2) as well as in subgroup (E1), in which the wounds were treated with silver sulfadiazine, the developed stratified squamous epithelium was covered with an eschar, under which there was a visible mature granulation tissue with a lot of fibers. In subgroup (E2), in which the wounds were treated with the bee pollen ointment, the whole wound surface was filled with a scar together with a thick stratified squamous epithelium. There was no granulation tissue. The E2 subgroup showed a correctly healed burn wound. The description of histopathological observations on 21st day of the experiment is shown in Figure 2.

### 3.3. Microbiological Test Results

#### 3.3.1. Quantitative Study

The Logarithmic CFU (colony forming unit) values of bacteria cultured on particular days of the burn wound healing are summarized in Table 1.

**Figure 3:**
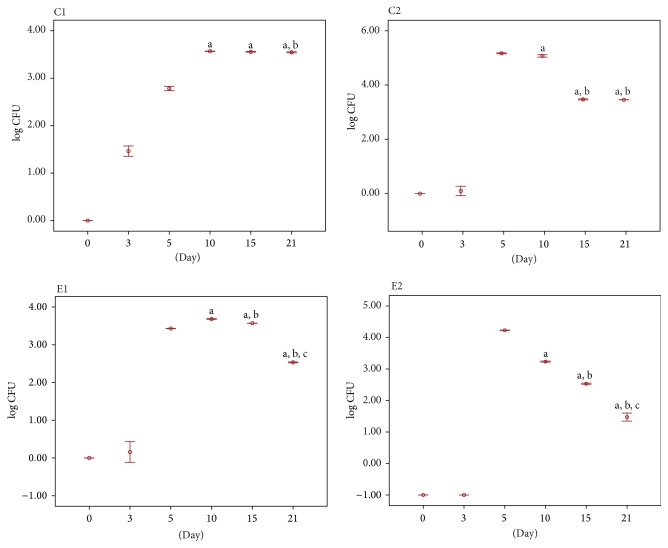
Quantitative study: log CFU value of bacteria cultured on particular days of the burn wound healing: C1: tissue material from untreated wounds; C2: tissue material collected from wounds washed with NaCl; E1: tissue material from wounds treated with silver sulfadiazine; E2: tissue material from wounds treated with bee pollen ointment. Results are expressed as mean ± standard error of the mean (SEM) of the assays performed in triplicate. ^a^ *p* < 0.05 compared with value determined on 5th day, ^b^ *p* < 0.05 compared with value determined on 10th day, and ^c^ *p* < 0.05 compared with value determined on 15th day.

**Figure 4:**
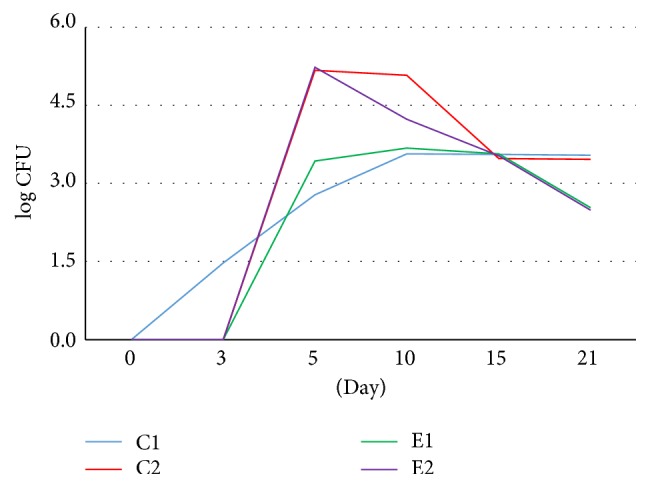
Dynamics of log CFU value of bacteria cultured on particular days of the burn wounds treated with NaCl (C2), silver sulfadiazine (E1), bee pollen ointment (E2), and untreated wounds (C1).

The result of the quantitative study conducted on the 0 day, immediately after burning, showed no microorganisms from none of the experimental groups. The effect of thermal feature made the skin sterilized. On the 3rd day of the study, the bacteria were isolated only from the tissue specimens collected from the untreated wounds. On the 5th day, the microorganisms were present in the tissue material of all studied groups. Further growth of the average number of bacteria in 1 cm^2^ of the wound was found on the 10th day of the experiment. However, the number of bacteria decreased in wounds washed with 0.9% of NaCl (C2) and in wound treated with the bee pollen ointment (E2). A further decrease of the number of bacteria in most analyzed groups was observed on the 15th day after burning. However, the wounds treated with the bee pollen ointment were characterized by the smallest number of bacteria in relation to the previous measurement. A systematic decrease of the number of bacteria in the wounds classifying to control and experimental groups was confirmed on the 21st day of the experiment and; what is more, the beds of thermal damage treated with silver sulfadiazine and with the bee pollen ointment were characterized by the biggest decrease of the bacteria number (Figures 3 and 4).

Logarithmic CFU (colony forming unit) values of fungi and mould cultured on particular days of the burn wound healing are summarized in Table 2.

**Figure 5:**
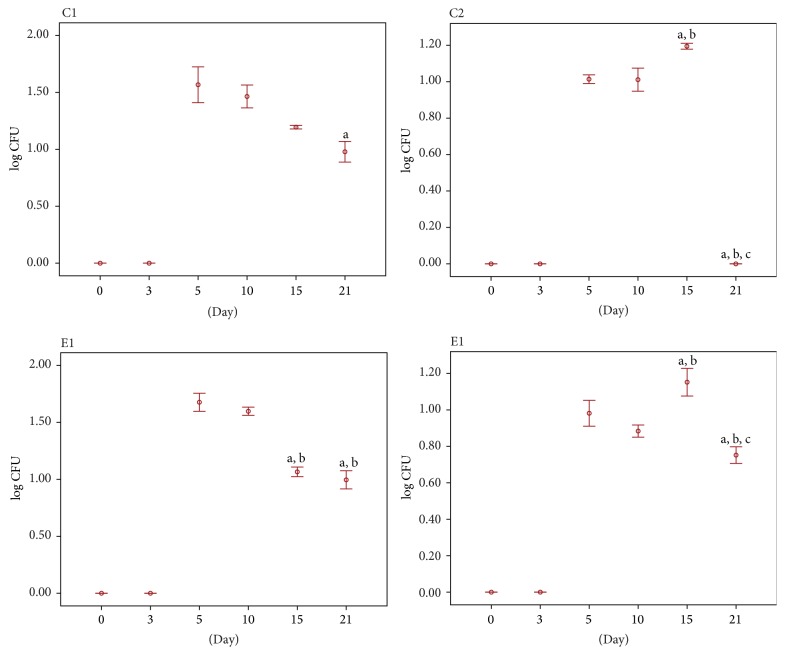
Quantitative study: log CFU value of fungi and mould cultured on particular days of the burn wound healing: C1: tissue material from untreated wounds; C2: tissue material collected from wounds washed with NaCl; E1: tissue material from wounds treated with silver sulfadiazine; E2: tissue material from wounds treated with bee pollen ointment. Results are expressed as mean ± standard error of the mean (SEM) of the assays performed in triplicate. ^a^ *p* < 0.05 compared with value determined on 5th day, ^b^ *p* < 0.05 compared with value determined on 10th day, and ^c^ *p* < 0.05 compared with value determined on 15th day.

**Figure 6:**
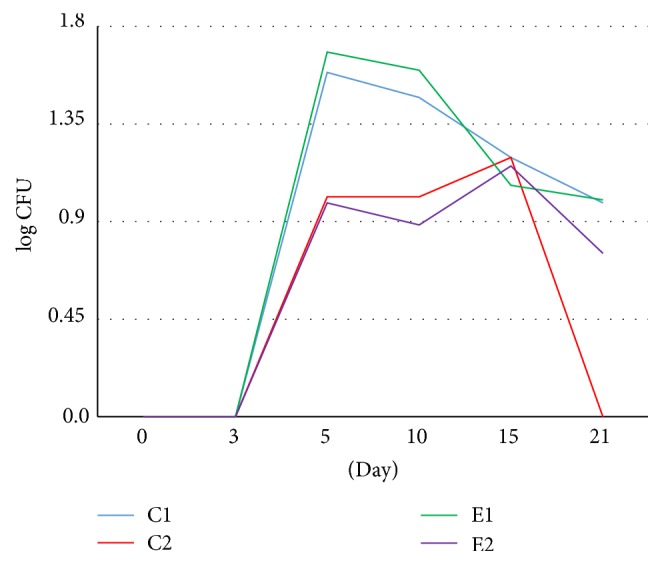
Dynamics of log CFU value of fungi and mould cultured on particular days of the burn wounds treated NaCl (C2), silver sulfadiazine (E1), bee pollen ointment (E2), and untreatedwounds (C1).

The growth of fungi and mould in the wound area of animals, evaluated on 0 and 3rd day of the C1, C2, E1, and E2 subgroups, resulted in finding no such microorganisms. The experimental studies conducted on 5th and 10th day showed that the number of fungi and moulds increased particularly in the case of untreated wounds as well as those treated with silver sulfadiazine. Next days showed a decreased general number of fungi and mould in untreated wounds and those treated with SSD. The wounds washed with NaCl and those exposed to bee pollen ointment were characterized by the lowest number of fungi and mould on the 21st day of the experiment (Figures 5 and 6).

Variable number of fungi and moulds in time was analytically analyzed. This analysis is to approximate the mode of changes in time with a model. A model, being the inertial first-order object with delay, has been chosen. The very choice of the model results from earlier authors' experiences concerning the analysis of dynamic changes (e.g., linked to temperature) occurring in humans and animals. The model enables parameterization of characteristics linked to the change rate of the number of fungi and moulds. These parameters are time constant *T*_1_ and delay. The time constant enables the determination of the change rate of the number of fungi and moulds in time. According to the theory of automatic control (the processes occurring in living organisms) the steady state takes place after third up to fifth time constants (95% and 99% of the steady state). For the cases in question it means that if the obtained results are approximated with this model (the inertial first-order object with delay) it will become possible to determine the time after which the decrease in the number of fungi and moulds to the values close to 0 (zero) will appear. It will be, for example, 3*∗T*_1_ for which only 5% of fungi and mould will remain when related to the maximum value. Similarly for 5*∗T*_1_ only 1% of fungi and mould will remain in relation to the maximum value. The approximation of changes of the number of fungi and mould in time with the inertial first-order object with delay enables obtaining one more error parameter of matching *δ*, which gives the information about the matching compliance of the model with the obtained experimental data.

The time change of the average number of bacteria, fungi, and moulds is a nonlinear relationship. Due to the analogy to the control systems, the response of the system (in this case it is the number of bacteria, moulds, and fungi) may be approximated by the inertial first-order object with delay. It results from the biological and medical rationale concerning the growth rate (development) of the bacteria, fungi, and moulds on the healing wound surface (regardless of the fact if it was C1, C2 or E1, E2). The general transmittance form of the response relationship for the multi-inertial object is as follows:

(1) $$\begin{array}{c} G\left(\;s\;\right)=\frac{k}{\left(1+sT_{1}\right)\left(1+sT_{2}\right)\cdots \left(1+sT_{n}\right)}, \end{array}$$

where *k* is amplification and *T*_1_, *T*_2_,…, *T*_*n*_ is time constant.

This was the basis for formulating the error of matching the model with the source data, for example, for the bacteria (superscript) and tissue material from untreated wounds (subscript) done as follows:

(2) $$\begin{array}{c} \delta_{C1}^{\left(B\right)}=\frac{100}{I\cdot \max_{i}y_{C1}^{\left(B\right)}\left(i\right)}\overset{I}{\underset{i=1}{\sum }}\left|\;y_{C1}^{\left(B\right)}\left(\;i\;\right)-y_{SC1}^{\left(B\right)}\left(\;i\;\right)\;\right|\left[\;\%\;\right], \end{array}$$

where *y*_C1_^(*B*)^(*i*) is change in the number of bacteria (superscript *B*) in the next *i*-measurements,  *y*_SC1_^(*B*)^(*i*) is simulation of change in the number of bacteria (superscript *B*) in the next *i*-measurements for the model (unit response) described by transmittance, and *I* is total number of measurements.

Similarly, the error of experimental data match with the standard for fungi and mould (superscript *R*) and different materials is calculated. For such a formulated error the method of a tuned model was applied in order to match the multi-inertial object with the data and to specify the order of the model. The smallest values of errors, shown in Table 3, were obtained for multi-inertial first-order object.

**Figure 7:**
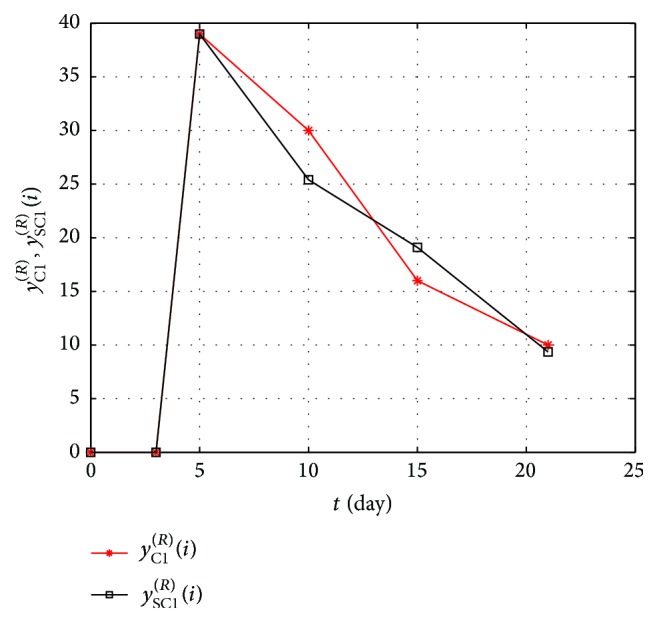
The graph of changes in the number of fungi and moulds in time for experimental and simulation data of the material C1.

In Table 3 the calculated error values of the match *δ* ^(*B*)^ and *δ* ^(*R*)^ for the materials C1, C2, E1, and E2 were shown. The calculations were done for the inertial first-order object (with the time constant *T*_1_) with delay (5 days) for which the value of particular errors is smaller. In the graph in Figure 7 the exemplary obtained results are shown, the behaviors of *y*_C1_^(*R*)^(*i*) and *y*_SC1_^(*R*)^(*i*) for *T*_1_ = 8.

As it can be concluded from Figure 7, the biggest error values (>44%) occur for materials C2 and E2, which results from the specification of changes in the number of bacteria in the wound. Due to individual variation of pigs, this specification depends on many factors. The smallest error values and, simultaneously, the best match of the model with experimental data occur for materials C1 and E1. The time constants for them are 20 and 18 days. Similar error values were obtained for fungi and mould which fluctuate around the value of 18%. The time constants are also different (as in the case of bacteria) for materials C1 and E1 amounting to 6 and 7 days, while for materials C2 and E2 they are equal to 20 days. Summing up the obtained results, the time constant average value of the growth of bacteria, mould, and fungi in the wound is at the level of 18 up to 20 days.

#### 3.3.2. Qualitative Study

In the qualitative study, changes of microbial species from the swabs of burn wounds treated with appropriate experimental agents and of the healthy skin surface were evaluated during next days of the experiment. On 0 day, the number of microorganisms, which constitute the physiological flora of the skin and the environment, increased in healthy skin (Table 4). However, no bacteria were cultured from none of the samples collected from the burn wounds immediately after burning.

On the 3rd day of the study, the wounds were colonized with microorganisms from* Micrococcus* species only in the subgroup in which the wounds were untreated (C1). On the 5th day of the study, the number of isolated microorganisms species significantly increased in the animals of all subgroups. Besides typical physiological flora of the skin and the environment (*Micrococcus* spp.,* Bacillus* spp.) there were also microorganisms which are characteristic for wound inflammation. In the subsequent days of the experiment, all burn wounds were characterized by a lower number of strains. On the 21st day of the study, in subgroups C2, E1, and E2, the bacterial flora was reduced to only one environmental species, such as* Bacillus* spp., while in the group of untreated wounds (C1), only* Staphylococcus hyicus* was found.

## 4. Discussion

Wound healing is a dynamic and time-synchronized reaction of the organism connected both with the actions of many cells, such as inflammatory cells, vascular cells, connective tissue cells, and epithelial cells, and with accumulating extracellular matrix (ECM) components, which leads to creation of a new tissue [20]. A significant role in the healing process is played by ECM components: glycosaminoglycans (GAGs), fibronectin, proteoglycans, vitronectin, and collagens [21, 22]. The therapeutic effect of a natural bee preparation, propolis, in the treatment of experimental burn wounds was the subject of our previous experimental studies. They showed that application of propolis modulated the expression of glycosaminoglycans, collagens, noncollagenous glycoproteins, and free radicals in the burn wound bed, which favors the intensification of healing process and, therefore, confirmed a positive influence of the mentioned apitherapeutic agent on the metabolism of ECM components [21–24].

The aim of the present study was to compare the therapeutic efficiency of another natural agent based on bee pollen extract with a commonly used pharmaceutical silver sulfadiazine in treatment of thermal burns.

Although silver sulfadiazine is considered as a gold standard in the topical treatment of burn wounds, this therapeutic agent is characterized by many side effects such as the risk of crystalluria, methaemoglobinaemia, neutropenia, erythema multiforme, and prolonged reepithelialization and the impairment of the mechanical strength of newly created tissue [25, 26]. Such side effect cannot be found in the case of bee pollen. This apitherapeutic agent demonstrates strong immune-modulating properties, which accelerate epithelialization and has bacteriostatic, bactericidal, and anesthetic properties [9, 27]. Moreover, bee pollen has a strong anti-inflammatory activity, decreases the healing period, and reduces the duration and intensity of ailments [9, 28].

The experimental model implemented in the present study was based on the tissue material collected from the domestic pig skin. The choice of the animal was made mainly due to the similarity between pig skin and human one [29].

Clinical and histopathological observation comprising the assessment of the extent and depth of the burn wounds, wound maceration, presence of necrotic tissue, granulation tissue type, and swelling around the burn wound indicated that, on the first days of the experiment, the pathomorphological view of the wounds for every group was the same. It became significantly differentiated on the 5th day of the observation. In the case of the wounds treated with the ointment with bee pollen ointment (E2), the wound area was covered with a thin, flexible eschar with a slight bleeding. In the wound area there were signs of swelling and reddening.

On the next days of the observation of the wounds treated with the apitherapeutic agent, a strong granulation and, subsequently, epithelium formation with clearly visible fully healed characteristics were noted. The wound surface decreased and was the size of 1 cm × 1 cm. In the area of the tissues surrounding the healing wound there were no signs of swelling or the ongoing inflammatory process. The clinical and histopathological assessment led to a conclusion that the applied apitherapeutic agent ointment reduces the time of burn wound treatment. Similar results were obtained in our previous studies where the therapeutic usability of another apitherapeutic agent, propolis, was assessed in the course of regeneration of experimental thermal skin damage. Propolis ointments in comparison with SSD preparation significantly accelerated the regenerative-reparative process of tissue damage not demonstrating any undesirable effects at the same time [30]. The beneficial effect of standardized propolis formulation on the healing process was also proved in Jastrzębska-Stojko et al. experimental studies [31]. The healing process of burn wounds treated with Sepropol was faster as compared to the standard SSD therapy. Moreover, histopathological tests showed that the process of scar formation in wounds treated with propolis formulation started considerably earlier as compared to the control group [31]. The other part of our studies concerning microbiological examinations during experimental burn wound healing proved that bee pollen ointment had an effective antimicrobial activity, reducing both the number of microorganisms and presenting bactericidal activity in isolated strains. The antibacterial properties of another apitherapeutic agent, propolis, were already assessed in the study with animal model of burn wounds. The mentioned study indicated a higher antimicrobial effectiveness of propolis ointment as compared to SSD in the course of burn wounds healing. A more beneficial action of the first from the mentioned preparations was manifested by a significant reduction of microorganisms as well as a more effective bactericidal action of the applied apitherapeutic agent. A similar trend in the effects of SSD action and a bee product in the range of antibacterial action were described by Kabała-Dzik et al. [32].

The therapeutic mechanism of bee natural products is based, among others, on antimicrobial activity and on inducing processes of damaged tissues regeneration. These characteristics proved their usability in wound healing and ulcerations of different etiology [31, 33].

The results in this study confirmed the beneficial effect of the bee pollen ointment on the burn wound healing process which could be seen in the decreased number of bacteria in the burn wounds during subsequent days of the experiment.

Different mechanisms could be responsible for the observed antibacterial effects of bee pollen. The first one results from the presence of active compounds, such as flavonoids and phenolic acids, whose forming complexes with bacterial cell walls lead to the disruption of cell wall integrity, blocking ion channels, and inhibiting electron flow in the electron transport chain [34].

The second mechanism by which bee pollen exerts antibacterial activity might be based on the inhibition of bacterial RNA-polymerase by phenolic compounds such as flavanone pinocembrin, flavonol galangin, and caffeic acid phenethyl ester [35].

Besides high antimicrobial activity, bee pollen ointment was also characterized by a bactericidal effect for isolated strains.

Moreover, the study also proved that thermal damage and bacterial infection of the wound favor yeast multiplication including* Candida albicans.* The yeast of the* Candida* species in proper conditions is saprophytes which live in the natural environment and colonize mucosa and human skin. However, they may induce life-threatening candidiases. Burns and necrotic lesions, which are the gates for fungal infection, may contribute to sepsis.* Bacillus cereus* and* Bacillus subtilis*, which are usually harmless, may induce infections in the condition of decreased immunity.

The clinical and histopathological observations performed in our study led to a conclusion that the bee pollen exerts a beneficial effect on wound healing cellular events providing reepithelization and wound closure. The microbiological studies proved that bee pollen ointment had an effective antimicrobial activity. The benefits and advantages of the bee pollen ointment in burn wound treatment imply the usability of the applied apitherapeutic agent preparation in topical burns therapy.

## Figures and Tables

**Table 1:** log CFU values of bacteria on the following days of the experiment.

	0 day	3rd day	5th day	10th day	15th day	21st day
C1 (untreated)	—	1.47	2.78	3.57	3.56	3.54
C2 (NaCl)	—	—	5.18	5.08	3.48	3.46
E1 (SSD)	—	—	3.43	3.68	3.57	2.53
E2 (bee pollen)	—	—	5.23	4.24	3.53	2.48

**Table 2:** log CFU values of fungi and mould on the following days of the experiment.

	0 day	3rd day	5th day	10th day	15th day	21st day
C1 (untreated)	—	—	1.59	1.47	1.19	0.99
C2 (NaCl)	—	—	1.01	1.01	1.19	0.18
E1 (SSD)	—	—	1.68	1.60	1.07	1.00
E2 (bee pollen)	—	—	0.99	0.88	1.16	0.75

**Table 3:** The error value of matching the inertial first-order object model with the experimental data (bacteria, fungi, and mould) for given time constants *T*_1_.

	C1	C2	E1	E2
*δ* ^(*B*)^ [%]	3	44	5	58
*T*_1_ [day]	20	1	18	1
*δ* ^(*R*)^ [%]	25	17	18	17
*T*_1_ [day]	7	20	6	20

**Table 4:** Changing of species of microflora in burn wounds in the following days of the experiment; N: tissue material from healthy skin not inflicted with a burn; C1: tissue material from untreated wounds; C2: tissue material from places washed with 0.9% NaCl; E1: tissue material from places treated with silver sulfadiazine salt; E2: tissue material from places treated with the with bee pollen ointment.

	0 day	3rd day	5th day	10th day	15th day	21st day
N	*Micrococcus* spp. *Bacillus* spp. *Staphylococcus lentus*	*Micrococcus* spp. *Bacillus* spp. *Staphylococcus lentus*	*Micrococcus* spp. *Bacillus* spp. *Gemella *spp. *Aerococcus viridans*	*Micrococcus* spp. *Bacillus* spp. *Aerococcus viridans*	*Micrococcus* spp. *Bacillus* spp. *Aerococcus viridans* *Enterococcus faecalis*	*Micrococcus* spp. *Bacillus* spp. *Aerococcus viridans*

C1	—	*Micrococcus* spp.	*Micrococcus* spp.	*Micrococcus* spp. *Staphylococcus hyicu*s *Candida* spp.	*Candida* spp.	*Staphylococcus hyicus *

C2	—	—	*Micrococcus* spp.	*Micrococcus* spp.	*Bacillus* spp.	*Bacillus* spp.

E1	—	—	*Bacillus* spp. *Staphylococcus hyicus* *Enterococcus faecalis*	*Bacillus* spp. *Micrococcus* spp.	*Bacillus* spp.	*Bacillus* spp.

E2	—	—	*Bacillus* spp. *Staphylococcus hyicus* *Pseudomonas aeruginosa*	*Bacillus* spp. *Pseudomonas aeruginosa*	*Bacillus* spp.	*Bacillus* spp.
